# A novel approach to interpret quasi-collimated beam results to support design and scale-up of vacuum UV based AOPs

**DOI:** 10.1016/j.wroa.2022.100158

**Published:** 2022-10-09

**Authors:** N. Kovoor George, B.A. Wols, D. Santoro, M. Borboudakis, K. Bell, W. Gernjak

**Affiliations:** a University of Girona, Plaça de Sant Domènec, 3, 17004 Girona, Spain; b Wetsus, European Center of Excellence for Sustainable Water Technology, Oostergoweg 9, 8911MA Leeuwarden, the Netherlands; c KWR Water Research Institute, Groningenhaven 7, 3433 PE Nieuwegein, the Netherlands; d Trojan Technologies, 3020 Gore Rd, London, ON N5V 4T7, Canada; e USP Technologies Canada ULC, 3020 Gore Rd, London, ON N5V 4T7, Canada; f Brown and Caldwell, Walnut Creek, CA94596 , California, United States; g Catalan Institute for Water Research (ICRA), 17003 Girona, Spain; h Catalan Institution for Research and Advanced Studies (ICREA), 08100 Barcelona, Spain

**Keywords:** Advanced oxidation processes, Organic micropollutants, Quasi-collimated beam, UV/H_2_O_2_, Vacuum ultraviolet

## Abstract

UV-C at 254 nm and vacuum UV (VUV) at 185 nm are the two major emission lines of a low-pressure mercury lamp. Upon absorption of VUV photons, water molecules and selected inorganic anions generate hydroxyl (HO^.^) and other redox radicals, both capable of degrading organic micropollutants (OMPs), thereby offering the opportunity to reduce H_2_O_2_ and energy consumption in UV-based advanced oxidation process (AOP). To be successfully scaled-up, the dual-wavelength VUV+UV/H_2_O_2_ AOP requires laboratory-scale experiments to establish design criteria. The figures of merit typically used for reporting and interpreting quasi-collimated beam results for UV-based AOPs (time, dose, absorbed energy and E_EO_) are insufficient and inaccurate when employed for dual-wavelength AOP such as the VUV+UV/H_2_O_2_ AOP, and do not support system scale-up. In this study, we introduce a novel figure of merit, useful absorbed energy (uAE), defined as fraction of absorbed energy that results in the generation of oxidative radicals. Here, results of quasi-collimated beam VUV+UV/H_2_O_2_ AOP experiments on four different water matrices are used to introduce 2D plots that employ both uAE_UV_ and uAE_VUV_ as a novel method to represent laboratory-scale experiments of VUV+UV/H_2_O_2_ AOP and demonstrate how the 2D plots sufficiently support scale-up of the AOP.

## Highlights

• Vacuum UV based AOPs are effective and potentially chemical-free AOPs.
• There is an absence of appropriate figure-of-merit to support scale-up of vacuum UV based AOPs.
• Useful absorbed energy is an appropriate quantity to assess lab-scale vacuum UV based AOPs.
• 2D plots with useful absorbed energy help designing and scaling-up of vacuum UV based AOPs.

## Graphical abstract

**Figure:**
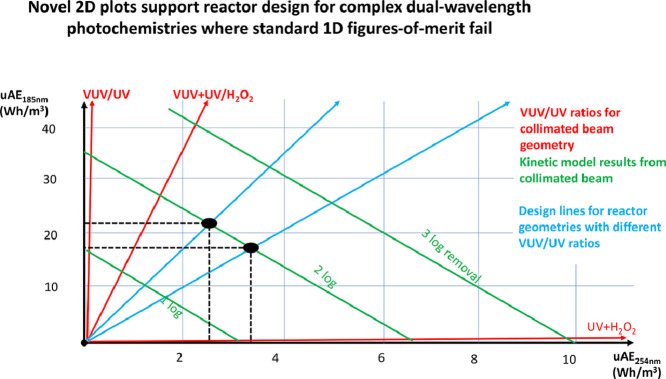

## 1 Introduction

Increasing use of pharmaceuticals, detergents, personal care products, fire-fighting agents, dyes and other anthropogenic chemicals to support modern life have resulted the introduction of these chemicals and their transformation products into the environment as micropollutants. Conventional biological treatment processes at (waste) water treatment plants are inadequate to remove many of these micropollutants (Capodaglio, 2020; Cuerda-Correa et al., 2020; Toor and Mohseni, 2007). Advanced oxidation processes (AOPs), however, have been proven to degrade the vast majority of organic micropollutants (OMPs) (Deng and Zhao, 2015) and are increasingly being incorporated as polishing steps in water treatment, particularly for applications of potable reuse (Cao et al., 2022; P. Sun et al., 2019).

UV-based AOPs employ UV radiation that generates radicals via photochemical reactions with oxidants. The oxidative radicals attack the OMPs either generating transformation products or completely mineralizing them into CO_2_ and H_2_O. The efficacy of an AOP in degrading OMPs depends on the photon and oxidative radical scavenging capacity of the water sample (Rosenfeldt and Linden, 2007) along with the design of the reactor.

UV/H_2_O_2_ AOP is one of the most extensively applied UV based AOPs (Zhan et al., 2021). UV photons at 254 nm (hereafter referred to as UV) from a low-pressure mercury (LP-Hg) lamp, generates HO^.^ via the homolysis of H_2_O_2_ molecule (Eq. (1)). Quantum yield, ,Φ in a photochemical reaction is defined as the amount of reactant consumed or product formed per photon absorbed. For the photolysis of H_2_O_2_ by UV, Φ=0.5 in terms of H_2_O_2_ consumed and Φ=1 in terms of HO^.^ produced because there are two HO^.^ produced per homolysis of H_2_O_2_ molecule.

(1) $$H_{2}O_{2}+h\nu_{254\text{nm}}\to 2HO^{.}\;\Phi =0.5$$

H_2_O_2_ is an inefficient absorber of UV because of its low molar absorption coefficient (20 M ^−^ ^1^.cm^−1^ (Weeks et al., 1963)). Therefore, only 5–10% of H_2_O_2_ that is dosed is utilized in most drinking water practices (Rosenfeldt et al., 2013). Consequently, quenching of H_2_O_2_ is needed, rendering UV/H_2_O_2_ AOP both energy and chemical intensive (Keen et al., 2013) and improvement of UV/H_2_O_2_ AOP is necessary for its broader use in the water industry.

The second major emission line of a LP-Hg lamp is at 185 nm (hereafter referred to as VUV). VUV is transmitted from the lamp when an appropriate lamp sleeve is used (Claus, 2021) and no additional electrical energy is required for its generation. VUV generates HO^.^ among other redox radicals via photolysis (Eq. (2)) and homolysis (Eq. (3)) of water molecules. Therefore, combining VUV and conventional UV AOP could reduce the H_2_O_2_ required, and thereby the chemical cost, to achieve degradation of OMPs. Contrary to the poor absorbance of UV by H_2_O_2_, in VUV AOP, *all* incident VUV photons are absorbed by water or other major absorbers to generate radicals. Thus, the inherent efficiency of VUV AOP is substantially higher than UV/H_2_O_2_ AOP. The HO^.^ degrades the OMPs to form transformation products.

(2) $$H_{2}O+h\nu_{<200\text{nm}}\to HO^{.}+\;H^{.}\;\Phi =0.33$$

(3) $$H_{2}O+h\nu_{<200\text{nm}}\to HO^{.}+\;H^{+}+{\;e}_{\text{aq}}^{-}\;\Phi =0.045$$

Due to the high absorption by pure water at 25 °C (1.76cm^−1^ (Weeks et al., 1963) at 55.55 M water concentration), more than 90% of emitted VUV photons are absorbed within 6 mm of the water surface. The absorbance in real water matrices in this study are even higher (Table 1). On the contrary, the penetration distance at which 90% of UV at 254 nm is absorbed can vary from 5 – 20 cm at UV transmittance (UVT) of 56% and 89% per cm, respectively. Optimizing a reactor for both the short penetrating VUV and relatively longer penetrating UV is challenging. Most of the laboratory-scale configurations, like *quasi*-collimated beam apparatus (*q*CB) (Baeza and Knappe, 2011; Duca et al., 2017; Fang et al., 2014; Feng et al., 2007), annular reactors (Ngouyap Mouamfon et al., 2011; Zhan et al., 2021; Zoschke et al., 2012) and micro-fluidic vacuum UV reactors (Yang et al., 2018) employed in VUV research have small mean reactor pathlengths that favorably uses VUV over UV. Despite extensive research published using laboratory-scale VUV reactors, there are scarce applications of VUV reported at pilot-scale (Krakko et al., 2021). Absence of a standard methodology to accurately represent the experimental *q*CB data and inform scale-up of VUV AOP stalls practical applications of this potentially chemical-free AOP. The performance of VUV+UV/H_2_O_2_ AOP depends on the design of the reactor, fluid hydrodynamics, water matrix and even the specific OMPs targeted for degradation. Such complexities are not accounted for in the simple figure of merits currently used (time (s), dose (mJ.cm^−2^), absorbed energy (kWh.m ^−^ ^3^), electrical energy per order (E_EO_, kWh.m ^−^ ^3^) etc.,) and consequently analysis based on these figures-of-merit risk under or over estimating the efficacy of VUV AOP. For example, time-based analysis of experiments performed in the aforementioned literature might lead to erroneous conclusion of a higher efficacy of VUV AOP than UV/H_2_O_2_ AOP because of the reactor design that favors the utilization of VUV. Therefore, development of a new methodology to represent and analyze laboratory-scale experimental results of VUV+UV/H_2_O_2_ AOP is warranted.

**Table 1:** Quality parameters of various water matrices experimented. RO-Reverse Osmosis permeate; DW_high Cl^−^: Drinking Water with High Cl^−^ concentration; DW_low Cl^−^: Drinking Water with Low Cl^−^ concentration; SWWE: Secondary Waste Water Effluent.

	unit	RO	DW_highCl^−^	DW_lowCl^−^	SWWE
TOC*/DOC**	mg.L ^−^ ^1^	< 1.00*	4.66*	3.24*	13.3**
IC	mg.L ^−^ ^1^	3.97	22	31.65	69.8
Turbidity	NTU	5.80E-02	0.11	0.16	281
COD	mg.L ^−^ ^1^	0.944	9.95	5.5	59.15
TSS	mg.L ^−^ ^1^	3.05	412	82	699
pH		6.23	7.13	8.01	8.1
Cl^−^	mg.L ^−^ ^1^	32.65	168	9.3	194
NO_3_^−^	mg.L ^−^ ^1^	3.3	1.33	0.74	<0.10⁎⁎⁎
SO_4_^−2^	mg.L ^−^ ^1^	0.6	64.2	0.79	41.65
HCO_3_^−^	mg.L ^−^ ^1^	8.22	94.5	157	346
CO_3_^−2^	mg.L ^−^ ^1^	5.61E-04	0.05	0.65	1.76
Br-	mg.L ^−^ ^1^	< 0.10	0.30	< 0.10	0.35
Na^+^	mg.L ^−^ ^1^	–	99.6	12.65	–
Ca^2+^	mg.L ^−^ ^1^	2.38	40.9	3.5	70.6
Mg^2+^	mg.L ^−^ ^1^	0.65	13.7	5.97	13.4
Hardness as CaCO_3_	mg.L ^−^ ^1^	8.62	158	33.2	231
absorbance UV	cm^−1^	0.00	0.05	0.05	0.53
absorbance VUV	cm^−1^	5.68	12.6	4.04	11.9
UV absorbed in 2 cm cell	%	2.7	20	20	91
VUV absorbed in 2 cm	%	100	100	100	100

⁎⁎⁎ The source of SWWE is a wastewater treatment plant with advanced biological treatment for nitrification/denitrification step with 92% total nitrogen removal. The exceptionally low NO_3_^−^ value of the SWWE is hypothesized to be due to an efficient functioning of the treatment step on the day of sampling.

In this paper, the merits and limitations of existing methodologies for interpreting laboratory-scale results are discussed. A novel figure-of-merit, useful absorbed energy (uAE) is introduced as an improvement to these limitations. Using experiments on various real water matrices, 2D plots employing uAE_UV_ and uAE_VUV_ is demonstrated as a new methodology for interpreting results of *q*CB experiments for VUV+UV/H_2_O_2_ AOP. These 2D plots can be used to predict the maximum degradation of targeted OMPs and predict the energy requirements in a VUV AOP, UV/H_2_O_2_ AOP and VUV+UV/H_2_O_2_ AOP thereby allowing *q*CB data to be used for scale-up of these AOPs. Additionally, the 2D plots inform the advantage of varying the UV to VUV output ratio of a lamp.

## 2 Materials and methods

### 2.1 Experimental reactor configurations

The *q*CB apparatus (see Fig. S1) consisted of a lamp housing and a collimator, both purged with nitrogen $\left(N_{2}\right)$ gas to prevent the formation of ozone $\left(O_{3}\right)$ which would otherwise be formed when VUV is absorbed by atmospheric oxygen $\left(O_{2}\right)$ (Oppenlaender, 2003). A 22.5 W low-pressure mercury lamp with major emission lines at UV and VUV was used. The distance between the center of the lamp and the surface of the sample was 33 cm. The distance was fixed based on a petri factor of 0.97 (Bolton and Linden, 2003). The diameter of the collimator was 5 ± 0.5 cm. The outlet of the collimator was equipped with a shutter which was closed after each irradiation experiment with an accuracy of ±5 s. A closed cylindrical quartz cell (type 35/Q, Starna Scientific, Germany) was filled with sample and placed directly under the collimator. A stir plate placed under the cell ensured the sample is well mixed. IL1700 radiometer employing the SED254/NS254 and SED185/NS185 detectors (International Light Technologies Inc., Massachusetts) were used to measure the UV and VUV irradiations respectively.

Three different advanced oxidation processes (AOP) were evaluated using the *q*CB setup. 1. VUV+UV/H_2_O_2_ AOP, 2. UV/H_2_O_2_ AOP and 3. VUV+UV AOP. In case of VUV+UV/H_2_O_2_ AOP and VUV+UV AOP, the sample with and without H_2_O_2_ respectively, was placed under the *q*CB setup and irradiated. To block VUV from reaching the sample in UV/H_2_O_2_ AOP experiments, a closed cylindrical quartz cell (1 cm) filled with 4 M NaCl was placed on top of the sample cell. This arrangement also resulted in a reduction of 23% of incident UV irradiation, which was accounted for in analyses of results.

### 2.2 Chemical analyses

Anions and cations were measured using ion chromatography using a Thermo Scientific Dionex Aquion with a Dionex Ionpac AS22 RFIC column, and Dionex Ionpac AG22 RFIC pre-column. Total (in)organic carbon was measured using a Shimadzu TOC-L TOC analyser. Liquid chromatography-mass spectrometry (LC-MS) was used to measure OMP concentrations. The LC was outfitted with an Agilent Zorbax Eclipse Plus C18 RRHD (1.8 μm, 50×2.1 mm) column, equipped with a UHPLC guard Zorbax Eclipse Plus C18 (1.8 μm, 2.1 × 5 mm) pre-column. The flow rate was set at 0.25 mL min^−1^ and the column temperature was maintained at 40 °C. For the MS, an Agilent 6420 Triple Quadrupole (QqQ) Mass Analyzer with electrospray ion source was used. Table 1 indicates the quality of various water matrices experimented in this study.

### 2.3 Determination of UV and VUV transmittance of samples

A Shimadzu UV-1800 spectrophotometer at 254 nm was used to measure UVT of the samples (see UV absorbance in Table 1). VUV transmittance was measured using the *q*CB setup. First, an empty 1 mm closed cylindrical quartz cell was placed on top of the detector. The shutter of the qCB setup was opened and the radiometer value noted. The procedure was repeated with the 1 mm closed cylindrical quartz cell filled with sample and the difference in intensities measured at the two conditions were used to calculate the absorbed intensity, from which absorbance was calculated (see Table 1). Beer-Lambert's law was used to calculate the transmittance from the absorbance.

### 2.4 Determination of irradiance at UV and VUV

Chemical actinometry methods were used to validate the output measured using the IL1700 radiometer employing the SED254/NS254 and SED185/NS185 detectors. Potassium iodide/potassium iodate (KI/KIO_3_) actinometry (Qiang et al., 2015) was used to measure the incident irradiance at UV. Probe scavenger actinometry (Furatian, 2017) was used to determine the VUV output. In this method, degradation kinetics of a probe in the presence of a defined amount of scavenging capacity under VUV irradiation is used to calculate the incident fluence rate at VUV. The quantum yield for water photolysis, second order rate constants for the probe compound and a scavenger with $HO^{.}$ must be known a priori. Both the probe and scavenger must only have a negligible direct photolysis rate and absorption at UV with no other significant degradation pathway other than oxidation via with $HO^{.}$.

### 2.5 Experimental design

To accentuate that the inaccuracy of the various figures-of-merit currently used is valid across various water matrices and OMPs, water matrices and OMPs of principally varying characteristics were chosen (Table 1). The four water matrices chosen had varying degrees of UVT (reverse osmosis permeate and secondary waste water effluent are respectively the best and worst case scenario water matrices for application of UV based treatments, given their respective high and low UVT), anion contents (two drinking water samples especially vary in their chloride content), organic HO^.^ scavenging capacity etc.,. Likewise, the five OMPs were chosen based on their varying degrees of direct photolysis at UV and second-order reaction rate constants with HO^.^ . $\left(k_{HO^{.}}\right)$ Table 2 classifies the OMPs based on the degree of direct photolysis and $k_{HO^{.}}$ into high, average and negligible.

**Table 2:** Photochemical constants at UV and second-order reaction rate constants with HO^.^ of the various OMPs used.

	Φ_254_ (mol.Einstein^−1^)	Ɛ_254_ (M ^−^ ^1^.cm^−1^)	Direct photolysis (Φ_254_ x Ɛ_254_) (L.cm^−1^.Einstein^−1^)	$k_{HO^{.}}$(M ^−^ ^1^.s ^−^ ^1^)
Atrazine	0.033 1	3.68E+03 1	121.44 Average	2.5e+09 2 Average
Carbamazepine	6.00±0.89e-04 3	6.07e+03 3	3.64 Negligible	9.4e+09 4 High
Diclofenac	0.272±0.046 5	4260±130 5	1158.72 High	7.5 ± 1.5e+09 6 High
Metformin		128 7	Negligible	1.4E+09 8 Low
n‑butyl paraben	0.0033±0.0004^9^	15,400^9^	50.82 Low	4.27±0.05E+09 10 Average

Note that discussion on specific degradation kinetics of OMPs, matrix effect on OMP degradation, comparisons of OMP degradation etc., were beyond the scope of this study. Additionally, no results regarding diclofenac are discussed because it could not be detected in the irradiated samples due to its extremely fast degradation.

1 (Bolton and Stefan, 2002).

2 (Nick et al., 1992).

3 (Pereira et al., 2007).

4 (Lam and Mabury, 2005).

5 (Canonica et al., 2008).

6 (Huber et al., 2003).

7 (Prasanth and Eapen, 2012).

8 (Błedzka et al., 2009).

9 (Wols et al., 2013).

10 (Błedzka et al., 2012).

### 2.6 Definitions of terminologies

The definitions of the major terminologies used in the results and discussion section are described in this section.

#### 2.6.1 Dose, D_λ_ (J.m ^−^ ^2^)

Dose, or fluence, is defined as the time integrated average irradiance (note that irradiance and fluence rate are the same in a collimated beam setup) received by the sample over a specific time (Eq. (4)).

(4) $$D_{\lambda }=\;I_{avg,\lambda }*t$$

(5) $$I_{avg,\lambda }=\left(I_{0,\lambda }*RF*PF*DF\right)*\frac{\left(1-10^{-a_{\lambda }l}\right)}{\ln\left(10\right)*a_{\lambda }*l}=\frac{I_{abs,\lambda }}{\ln\left(10\right)*a_{\lambda }*l}$$

Where, $D_{\lambda }$ is the dose at a given wavelength, *λ*, in J.m ^−^ ^2^; $I_{avg,\lambda }$, $I_{0,\lambda }$ and $I_{abs,\lambda }$ are the average irradiation delivered to the sample, irradiation incident on the sample surface and irradiation absorbed by the sample in W.m ^−^ ^2^ at a given wavelength respectively; t is the time for which the sample was irradiated in s; RF, PFandDFare the reflection factor, petri factor and divergence factor respectively and the term $\frac{\left(1-10^{-a_{\lambda }l}\right)}{\ln\left(10\right)*a_{\lambda }*l}$ represents the water factor (Bolton and Linden, 2003); $a_{\lambda }$ is the absorbance of the sample at a given wavelength in m ^−^ ^1^; l is the pathlength of the sample in m. The values of RF, PF and DF are calculated separately for each water type and wavelength. Note that in a *q*CB system, mJ.cm^−2^, mW.cm^−2^, cm^−1^ and cm are the commonly used units for dose, irradiation, absorbance and path length.

#### 2.6.2 Absorbed energy, AE_λ_ (Wh.m ^−^ ^3^)

Not all incident irradiation entering the fluid domain is absorbed by the sample. The fraction of irradiation, at a given wavelength, that a sample can absorb depends on the absorbance of the sample at that wavelength. The absorbed energy (AE) of a sample is defined as the fraction of irradiation absorbed by the sample (Eq. (6)).

(6) $$AE_{\lambda }=\frac{I_{abs,\lambda }}{3600}*\frac{S}{V}*t$$

Where, S is the surface area of the sample irradiated in m^2^; V is the volume of the sample irradiated in m^3^. (Note that $\frac{S}{V}$ is equal to the pathlength l of the sample in case of the cylindrical cell used in this study). The factor $\frac{1}{3600}$ converts J.m ^−^ ^2^ to Wh.m ^−^ ^2^. Incorporating Eq. (4) and Eq. (5) in Eq. (6), results in Eq. (7).

(7) $$AE_{\lambda }=\frac{D_{\lambda }*\ln\left(10\right)*a_{\lambda }}{3600}$$

#### 2.6.3 Useful absorbed energy, uAE_λ_ (Wh.m ^−^ ^3^)

Useful absorbed energy (uAE) is the fraction of AE that enters the fluid and is absorbed by constituents in the water sample to generate radicals that contribute towards degradation of OMPs. For example, in this study, UV photons absorbed by the organic matter of the sample is assumed to be a wasted energy as compared to that absorbed by H_2_O_2_ because UV absorption by the former does not result in the formation of radicals that are capable of degrading OMPs. The uAE_λ_ of a sample is obtained using Eq. (8). By combining Eqs. (5), Eq. (6)and Eq. (8), uAE_λ_ in terms of incident irradiation is obtained.

(8) $$uAE_{\lambda }=AE_{\lambda }*\;f_{\lambda }$$

(9) $$uAE_{\lambda }=\frac{I_{abs,\lambda }}{3600}*\frac{S}{V}*t*f_{\lambda }=\left(I_{0,\lambda }*RF*PF*DF\right)*\frac{\left(1-10^{-a_{\lambda }l}\right)}{3600}*\;\frac{S}{V}*t*f_{\lambda }$$

Where $f_{\lambda }$ is,

(10) $$f_{UV}=\;\frac{\varepsilon_{H_{2}O_{2},UV}*C_{H_{2}O_{2}}+\varepsilon_{x,UV}*C_{x}}{\sum_{i}^{n}\varepsilon_{i,UV}*C_{i}}$$

(11) $$f_{VUV}=\frac{\varepsilon_{H_{2}O,VUV}*C_{H_{2}O}+\varepsilon_{H_{2}O_{2},VUV}*C_{H_{2}O_{2}}+\varepsilon_{x,VUV}*C_{x}}{\sum_{i}^{n}\varepsilon_{i,VUV}*C_{i}}$$

Where $\varepsilon_{i,\lambda }$ is the molar absorbance of constituent i in sample at a given wavelength in M ^−^ ^1^.m ^−^ ^1^ and C is its respective concentration in M; the term $\frac{\varepsilon_{x,\lambda }C_{x}}{\sum_{i}^{n}\varepsilon_{i,\lambda }*C_{i}}\;$includes the fraction absorbed by any other constituent x that can generate oxidative radicals at a given λ; n is the number of constituents in the sample. Note that in a UV/H_2_O_2_ AOP, H_2_O_2_ will, in most cases, be the only constituent that contributes to generation of radicals on absorption of UV. With respect to VUV, besides H_2_O and H_2_O_2_, some anions like chloride (Cl^−^), nitrate (NO_3_^−^) and sulfate (SO_4_^2−^) could also absorb VUV photons to produce oxidative radicals. For the sake of simplicity, AE_VUV_ is considered equal to uAE_VUV_ in this study, in other words, all the absorbed energy at VUV results in the generation of useful radicals. The validity of the assumption is further elaborated in Section 3.4.

#### 2.6.4 Electrical energy per order, E_EO_ (Wh.m ^−^ ^3^)

E_EO_ is defined as the energy required to reduce the concentration of an OMP by 1-log in 1 m^3^ of contaminated water.

(12) $$E_{EO}=\frac{P}{\dot{Q}*log_{10}\left[\frac{C_{i}}{C_{0}}\right]}$$

Where P is the total electrical power of the lamps employed in W; and$\dot{Q}$ is the flow rate of the fluid in m^3^.h ^−^ ^1^; $log_{10}\left[\frac{C_{i}}{C_{0}}\right]$ is the log degradation of the contaminant where $C_{i}$ and $C_{0}$ are the final and initial concentrations of the contaminants. It is important to note that $E_{EO}$ is not a valid figure of merit for *q*CB setup rather it is a quantity that is used to compare the efficiency of different AOPs and reactors that are optimized for specific applications.

(13) $$E_{EO}=\frac{uAE_{\lambda }}{\log\deg of\;OMP*f_{\lambda }\eta_{\lambda }}$$

(14) $$E_{EO}=\frac{AE_{\lambda }}{\log\deg of\;OMP*\eta_{\lambda }}$$

Where, $\eta_{\lambda }$ is the efficiency of a lamp to convert the input electrical energy to UV or VUV output. Note that Eq. (13) and Eq. (14)are valid only when all the input energy at UV and VUV is absorbed by the sample.

## 3 Results and discussion

The results of the degradation studies on metformin (MTF), n‑butyl paraben (n-PBN), atrazine (ATZ) and carbamazepine (CBZ) in RO, SWWE, DW_high Cl^−^ and DW_low Cl^−^ water matrices in three different AOPs are discussed in this section. Various pathways of OMP degradation involved in each of these AOPs are described in Table 3.

**Table 3:** Various degradation pathways involved in VUV+UV/H_2_O_2_ AOP, UV/H_2_O_2_ AOP and VUV+UV AOP.

	VUV+UV/H_2_O_2_	UV/H_2_O_2_	VUV+UV
Degradation pathways			
Direct photolysis at VUV	X		X
Direct photolysis at UV	X	X	X
Oxidation by HO^.^ generated from the VUV/H_2_O	X		X
Oxidation by HO^.^ generated from the VUV/ H_2_O_2_	X		
Oxidation by HO^.^ generated from the UV/ H_2_O_2_	X	X	
Oxidation/Reduction by radical species formed from the matrix in combination with UV and VUV	X	X	X

General conclusions on the efficacy of AOPs vary depending on the k’ chosen. Data in Table 4 indicates the dependence of the ratios of k’_VUV+UV/H2O2_ / k’_UV/H2O2_ and k’_VUV+UV_ / k’_UV/H2O2_ for ATZ, CBZ, MTF and n-PBN in various water matrices on the choice of k’ (for absolute values of k’_time_, k’_total dose_, k’_total AE_ and k’_total uAE_, see Table S2). Note that ${k^{\prime }}_{\text{total}\;\text{dose}}=\frac{{k^{\prime }}_{\text{time}}*t}{\text{total}\;\text{dose}};\;{k^{\prime }}_{\text{total}\;\text{AE}}=\frac{{k^{\prime }}_{\text{time}}*t}{\text{total}\;\text{AE}}$; ${k^{\prime }}_{\text{total}\;\text{uAE}}=\frac{{k^{\prime }}_{\text{time}}*t}{\text{total}\;\text{uAE}}$. Also note that the units of the apparent reaction rate constants are given in Table 4, however, the ratios of k’_VUV+UV/H2O2_ / k’_UV/H2O2_ and k’_VUV+UV_ / k’_UV/H2O2_ are dimensionless.

**Table 4:** Ratios of k’_VUV+UV/H2O2_ / k’_UV/H2O2_ and k’_VUV+UV_ / k’_UV/H2O2_ for ATZ, CBZ, MTF and n-PBN in RO, DW_high Cl^−^, DW_low Cl^−^ and SWWE water matrices.

**Water type RO**	**k’**_**VUV+UV/H2O2**_**/ k’**_**UV/H2O2**_				**k’**_**VUV+UV**_**/ k’**_**UV/H2O2**_			

	ATZ	CBZ	MTF	n-PBN	ATZ	CBZ	MTF	n-PBN
k’_time_ (s ^−^ ^1^)	4.13	5.41	5.86	5.32	4.49	5.20	3.82	6.26
k’_total dose_ (mJ^−1^.cm^2^)	3.22	4.22	4.58	4.15	3.49	4.04	2.97	4.86
k’_total AE_ (W ^−^ ^1^.h ^−^ ^1^.m^3^)	0.21	0.28	0.30	0.28	0.24	0.28	0.21	0.34
k’_total uAE_ (W ^−^ ^1^.h ^−^ ^1^.m^3^)	0.19	0.24	0.27	0.24	0.22	0.25	0.18	0.30

**Water type** **DW_highCl-**	**k’**_**VUV+UV/H2O2**_**/ k’**_**UV/H2O2**_				**k’**_**VUV+UV**_**/ k’**_**UV/H2O2**_			

	ATZ	CBZ	MTF	n-PBN	ATZ	CBZ	MTF	n-PBN
k’_time_ (s ^−^ ^1^)	1.80	4.34	3.31	8.82	1.47	3.33	1.83	10.33
k’_total dose_ (mJ^−1^.cm^2^)	1.79	4.32	3.30	8.78	1.45	3.28	1.79	10.16
k’_total AE_ (W ^−^ ^1^.h ^−^ ^1^.m^3^)	1.03	2.49	1.90	5.06	0.88	2.00	1.09	6.18
k’_total uAE_ (W ^−^ ^1^.h ^−^ ^1^.m^3^)	0.21	0.50	0.38	1.02	0.19	0.44	0.24	1.35

**Water type** **DW_lowCl-**	**k’**_**VUV+UV/H2O2**_**/ k’**_**UV/H2O2**_				**k’**_**VUV+UV**_**/ k’**_**UV/H2O2**_			

	ATZ	CBZ	MTF	n-PBN	ATZ	CBZ	MTF	n-PBN
k’_time_ (s ^−^ ^1^)	3.83	7.43	11.50	6.37	2.87	4.83	9.13	3.93
k’_total dose_ (mJ^−1^.cm^2^)	2.81	5.46	8.44	4.68	2.09	3.52	6.66	2.86
k’_total AE_ (W ^−^ ^1^.h ^−^ ^1^.m^3^)	1.00	1.95	3.02	1.67	0.78	1.31	2.48	1.07
k’_total uAE_ (W ^−^ ^1^.h ^−^ ^1^.m^3^)	0.17	0.34	0.52	0.29	0.14	0.23	0.44	0.19

**Water type** **SWWE**	**k’**_**VUV+UV/H2O2**_**/ k’**_**UV/H2O2**_				**k’**_**VUV+UV**_**/ k’**_**UV/H2O2**_			

	ATZ	CBZ	MTF	n-PBN	ATZ	CBZ	MTF	n-PBN
k’_time_ (s ^−^ ^1^)	2.58	62.22	9.43	7.40	2.34	135.43	8.80	6.07
k’_total dose_ (mJ^−1^.cm^2^)	1.32	31.73	4.81	3.78	1.19	68.69	4.46	3.08
k’_total AE_ (W ^−^ ^1^.h ^−^ ^1^.m^3^)	1.02	24.48	3.71	2.91	0.92	53.52	3.48	2.40
k’_total uAE_ (W ^−^ ^1^.h ^−^ ^1^.m^3^)	0.12	2.82	0.43	0.34	0.11	6.51	0.42	0.29

The merits and limitations associated with reporting *q*CB results on the basis of the time, D_λ_ and AE_λ_ are discussed below. Subsequently, uAE_λ_ is introduced as a suitable figure-of merit which when implemented on a 2D plot supports the scale-up of AOPs.

### 3.1 Merits and limitations of using time as the figure of merit in reporting *q*CB results

The time based apparent reaction rate constant, k’_time_ (s ^−^ ^1^) and time (s) are frequently used for reporting of *q*CB experimental results of UV-based AOPs. For example, Guo et al., 2018 compared UV/chlorine and UV/H_2_O_2_ AOPs using k’_time_. Several experimental investigations also use on k’_time_ analysis of laboratory-scale experiments to compare VUV and UV AOPs, in most cases concluding VUV AOPs to have higher degradation efficacy than their UV counterparts (Fu et al., 2020; He et al., 2021; Y. Sun et al., 2019). However, such conclusions are only valid for specific laboratory-scale reactors employed and should not be generalized because k’_time_ do not account for effects of reactor design on utilization of UV and VUV in a reactor.

**Fig. 1:**
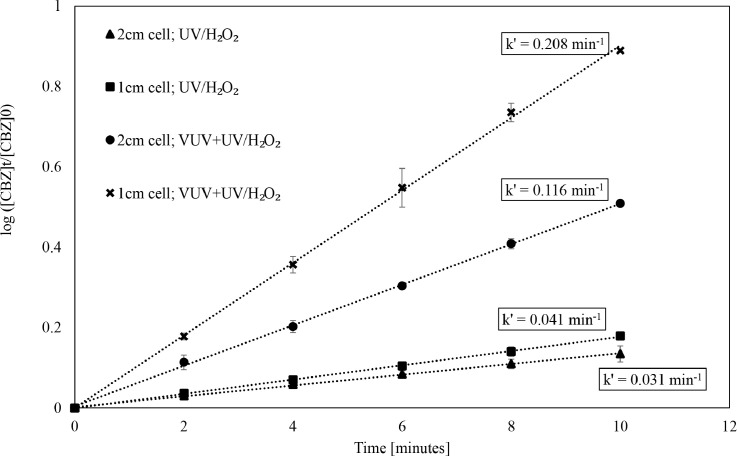
Log degradation of carbamazepine in MilliQ® water with tert‑butanol as scavenger and 7 mg.L ^−^ ^1^ H_2_O_2_ dosed. Comparison of degradation in a 1 cm cell versus 2 cm cell at various time using UV/H_2_O_2_ and VUV+UV/H_2_O_2_ AOP.

In the case of UV and VUV AOPs, the extreme difference in their penetration depths affects their relative contributions towards OMP degradation. For example, relative efficacies of VUV+UV/H_2_O_2_ AOP and UV/H_2_O_2_ AOP were compared based on k’_time_ using the experimental results of CBZ degradation in a sample of MilliQ®water with tert‑butanol as a scavenger after 10 min of irradiation in a 1 cm and 2 cm cell (Fig. 1). The ratio k’_time,VUV+UV/H2O2_/k’_time,UV/H2O2_ obtained from these experiments was 5.1 and 3.7 in a 1 cm cell and 2 cm cell, respectively. Thus, depending on pathlength, conclusions regarding the effect of combining VUV and UV/H_2_O_2_ AOP changes drastically when using k’_time_. In case of real water matrices, Table 1 indicates that in a 2 cm cell, *barely* 20% of the incident UV, (with the exception of SWWE) as compared to 100% of the incident VUV, is absorbed. In 1 cm cell the percent of the incident UV absorbed is further lower than in a 2 cm cell, however, the percent of the incident VUV absorbed remains 100% for all the water matrices. These results prove that reactor design (pathlength) favors the utilization of VUV. Therefore, when the results of OMP degradation in *qCB* experiments performed in this study using 1 cm or 2 cm cell are analysed based on k’_time_, both VUV+UV/H_2_O_2_ and chemical-free VUV AOP appears to have *higher* efficacy than the conventional UV/H_2_O_2_ AOP (k’_VUV+UV/H2O2_ / k’_UV/H2O2_ >1 and k’_VUV+UV_ / k’_UV/H2O2_ >1) for all water types and OMPs (Table 4). On the contrary, the conclusion drawn when the same *qCB* results are reported based on k’_total uAE_ is that both VUV+UV/H_2_O_2_ and chemical-free VUV AOP appears to have *lower* efficacy than the conventional UV/H_2_O_2_ AOP (k’_VUV+UV/H2O2_ / k’_UV/H2O2_ <1 and k’_VUV+UV_ / k’_UV/H2O2_ <1) for all water types and OMPs (except CBZ in SWWE).

Additional to the disregard to effects of reactor design, k’_time_ based results has two disadvantages: 1.k’_time_-based results cannot be reproduced or compared among different laboratories because it gives no indication of the total energy used by the system and 2. k’_time_ is inappropriate to represent results of both *q*CB and flow-through reactors (Zhan et al., 2021) because it does not indicate the total degradation time spent achieved in a reactor.

### 3.2 Merits and limitations of using dose (D_λ_) as the figure of merit in reporting *q*CB results

Because dose is calculated using the average irradiance a sample receives, it accounts for the pathlength of the sample (Eq. (4)), thereby overcoming the first drawback that time based assessment poses (see Section 3.1). Nonetheless, it must be noted that both D_UV_ and D_VUV_ are impacted by pathlength and absorptivity of the sample (see Fig. S2 in SI). 1D graphs representing D_total_ (or photon-fluence) versus log degradation of the OMP are currently employed to represent laboratory-scale results of AOPs (Li et al., 2019). Dubowski et al., 2020 and Moradi and Moussavi, 2018 used plots of D_UV_ vs. OMP degradation to represent degradation in a VUV+UV AOP. However, there are two major drawbacks in analyzing or comparing VUV and UV based AOPs using this approach.

First, VUV and UV based AOPs differ considerably in their inherent efficiency to generate HO^.^ proportional to photon dose, and their contributions towards the OMP degradation are mostly non-additive (Li et al., 2016; Sakai et al., 2021).

**Fig. 2:**
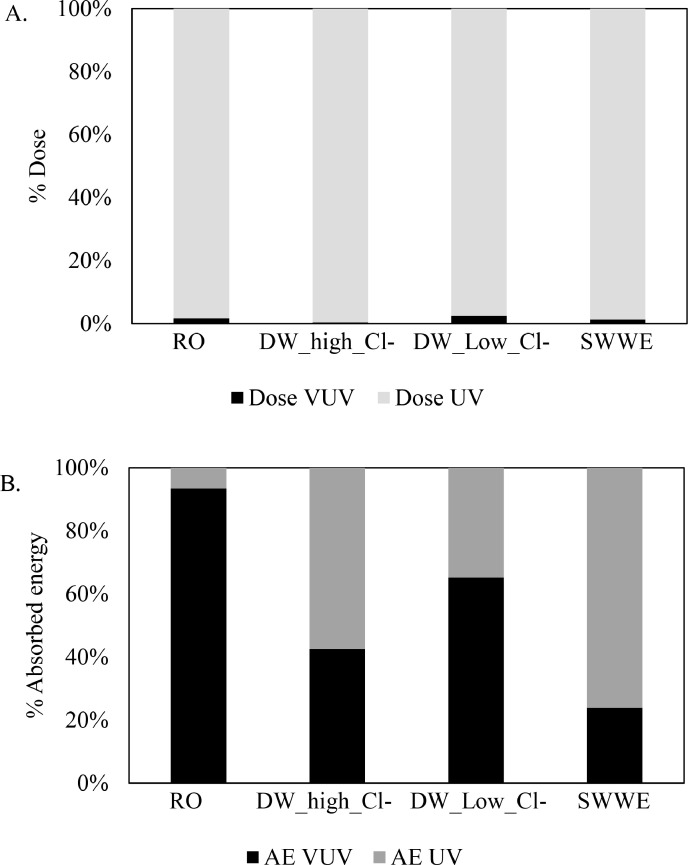

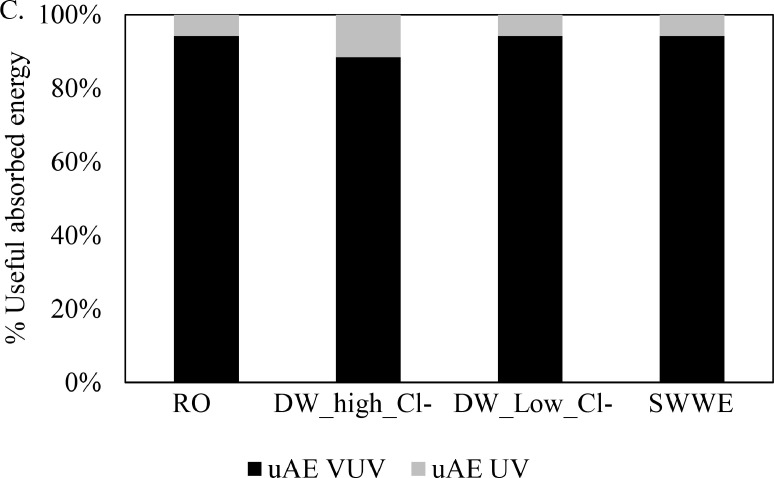
Percentage contributions of A. D_VUV_ and D_UV_ to the D_total_; B. AE_VUV_ and AE_UV_ to the AE_total_; and C. uAE_VUV_ and uAE_UV_ to the uAE_total_ in a 1 cm cell in RO, DW low Cl- and SWWE and in a 2 cm cell in DW high Cl- after 10 min of irradiation in VUV/UV/H_2_O_2_ AOP with [H_2_O_2_]=10 mg.L ^−^ ^1^.

Second, D_UV_ is the major contributor to D_total_ in the system (see Fig. 2), rendering D_total_ for a given sample in VUV+UV, VUV+UV/H_2_O_2_ and UV/H_2_O_2_ AOPs as approximately the same. D_UV_ is the major contributor because of the substantially higher UV output of LP-Hg lamps ($\eta_{\mathrm{UV}}$ = 25–40% and $\eta_{\mathrm{VUV}}\;$= 6–11%; (An et al., 2015; Schalk et al., 2005)) and the extremely low penetration depth of VUV compared to UV. For example, in the RO sample, VUV+UV AOP (in the absence of H_2_O_2_), D_UV_ does not contribute to degradation of CBZ (see Table 5) due to negligible direct photolysis at UV. CBZ degrades solely due to HO^.^ reactions generated via VUV AOP. Comparing the observed log degradations of CBZ in the various AOPs in RO water matrix (see Table 5), it is evident that VUV+UV AOP is highly efficient in degrading CBZ. However, the efficiency of VUV+UV AOP is underestimated (k’_VUV+UV_ / k’_UV/H2O2_ = 4.04, see k’_VUV+UV_ / k’_UV/H2O2_ using k’_total dose_ for CBZ degradation in RO water matrix in Table 4) when k’_total dose_ is used (where k’_total dose_ is 60 times smaller than k’_VUV dose_,see Table 5).

**Table 5:** D_UV_, D_VUV_, D_total_ received by RO water sample after 10 min of irradiation along with the actual log degradation of carbamazepine (CBZ) under various AOP settings contrasted against the apparent reaction rate constants, k’_UVdose_, k’_VUVdose_, k’_totaldose_.

	Dose received, D_λ_ (mJ.cm^−2^)			Log degradation of CBZ	Apparent dose-based reaction rate constant (mJ^−1^.cm^2^)		
AOP	D_UV_	D_VUV_	D_total_		k’_UVdose_	k’_VUVdose_	k’_totaldose_
UV/H_2_O_2_	121.3	0	121.3	0.4	7.7e-03	NA	7.7e-03
VUV/UV	153.2	2.6	155.8	2.7	3.2e-02	1.86	3.1e-02
VUV+ UV/H_2_O_2_	152.5	2.6	155.1	2.2	3.3e-02	1.94	3.3e-02

**Fig. 3:**
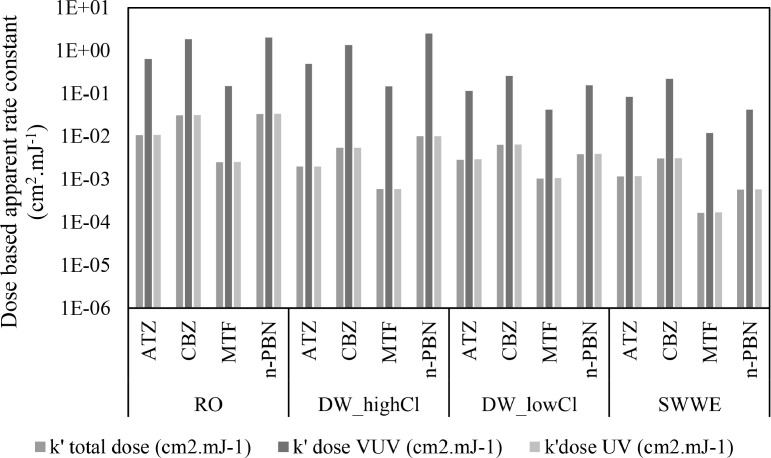
Comparison of k’_total dose_, k’_UV dose_ and k’_VUV dose_ of atrazine (ATZ), carbamazepine (CBZ), metformin (MTF) and n‑butyl paraben (n-PBN) in various water types using VUV+UV AOP. Experiments were performed in a 2 cm pathlength cell for DW_highCl sample and the rest in 1 cm pathlength cell.

Using both k’_UV dose_ and k’_VUV dose_ (or D_UV_ and D_VUV_) is a better approach than using k’_total dose_ or (D_total_). Gilboa et al., 2021 uses both D_UV_ and D_VUV_ to represent H_2_S degradation using VUV/UV AOP. However, from a scientific perspective, k’_UV dose_ and k’_VUV dose_ (or D_UV_ and D_VUV_) are difficult to compare because they differ by one or two orders of magnitude (see Fig. 3). From a practical perspective, the units of k’_UV dose_ and k’_VUV dose_ cm^2^.mJ^−1^(or D_UV_ and D_VUV_) do not intuitively support scale-up because there is no direct indication of the energy applied. The efficiency of an AOP to convert the applied dose to oxidative radicals is obscured from dose based figures-of merits. Note also that the efficiency of both VUV+UV AOP and UV/H_2_O_2_ AOP can significantly be affected by the amount of H_2_O_2_ dosed in to the sample (Li et al., 2019; Sakai et al., 2021). For example, k’_VUV+UV/H2O2_ / k’_UV/H2O2_ = 5.46 and k’_VUV+UV_ / k’_UV/H2O2_ = 3.52 for CBZ in DW_low_Cl- water matrix (see Table 4) when compared based on k’_total dose_. It is apparent that VUV+UV/H_2_O_2_ AOP has higher CBZ degradation efficiency than VUV+UV AOP at a given applied dose. However, the fact that the higher efficiency of the VUV+UV/H_2_O_2_ AOP is because of H_2_O_2_ dosing and thereby comes with additional operational cost, is not included in the dose based apparent reaction rate constants.

In summary, 1.use of k’_total dose_ or D_total_ is not accurate, 2. use of both D_UV_ and D_VUV_ obscures the inherent efficiencies of the various AOPs, and 3. use of both k’_UV dose_ and k’_VUV dose_ does not inform scale-up of the AOP. This demands a much more reliable figure of merit for novel AOPs to be more widely implemented in the water industry.

### 3.3 Merits and limitations of using absorbed energy (AE_λ_) as the figure of merit in reporting *q*CB results

AE_λ_ is based on the absorbance of a specific sample at a specific wavelength (see Section 2.6.2). AE_λ_ has complex relationships with the UVT_λ_ of the sample and the pathlength of the reactor (see Figure S3 in SI). Therefore, depending on the water matrix, either AE_UV_ or AE_VUV_ is the major contributor to AE_total_ (see Fig. 2). Due to the difference in the inherent efficiency of VUV AOP and UV/H_2_O_2_ AOP, the contributions of AE_UV_ and AE_VUV_ to OMP degradation are non-additive (see Section 3.1). Therefore, a plot of k’_AE,total_ (or AE_total_) versus log degradation of the OMP would not be sufficiently informative, because it is critical to define the relative contributions of k’_AE,UV_ and k’_AE,VUV_ (or AE_UV_ and AE_VUV_).

Employing a 2D plot with both k’_AE,UV_ and k’_AE,VUV_ still does not contribute to the scaling-up of the VUV+UV/H_2_O_2_ AOP. This is because the inherent efficiency of the VUV AOP and UV/H_2_O_2_ AOP in generating oxidative radicals is not deducible from AE_λ_. For example, in the case of the DW_highCl- sample, where both AE_UV_ and AE_VUV_ contribute roughly equally to AE_total_ (Fig. 2), only about 8.3% of the AE_UV_ is absorbed by H_2_O_2_, contributing to generation of oxidative radicals. Note that although the percent of UV absorbed by H_2_O_2_ can be increased with increasing H_2_O_2_ concentration, in the ranges that are relevant to industrial applications (7–20 mg.L ^−^ ^1^), the percent UV absorbed by H_2_O_2_ is not more than 25%. AE_VUV_ on the other hand, is completely absorbed in any practical reactor with a real water matrix (note that >90% of VUV photons are absorbed with 6 mm in double distilled water) and in most cases all the AE_VUV_ contributes towards generating oxidative radicals. It is thus warranted to introduce an appropriate figure of merit indicative of the inherent efficiency of various AOPs involved and assist in the scale-up of AOPs.

### 3.4 Useful absorbed energy (uAE_λ_) as an appropriate figure of merit in reporting *q*CB results

In a well-mixed reactor, the efficiency of an AOP in generating radicals is proportional to its capacity to degrade OMPs. Oxidative radical concentration is the product of AE_λ_, fraction of AE_λ_ absorbed by constituents that can generate oxidative radicals and the quantum yield of oxidative radicals. Various AOPs generate diverse oxidant radicals with compound-specific second order reaction rate constants. Based on their reactivity toward a specific OMP, the diverse oxidant radical concentration can be translated in to an equivalent HO^.^ concentration. Note that such an estimated HO^.^ density is OMP specific. The HO^.^ density generated via various AOPs can be used to compare their inherent efficiencies. However, such calculations require prior knowledge of quantum yield of oxidative radicals, which are not widely available. uAE_λ_ (the product of AE_λ_ and the fraction of AE_λ_ absorbed by the constituents that can generate oxidative radicals), can be a simple surrogate of oxidant concentration without jeopardizing its functionality.

In the calculation of uAE_UV_, H_2_O_2_ can be assumed to be the only constituent that generates radicals upon UV absorption. This is a valid assumption in the water matrices tested in this study because of the negligible direct UV photolysis of other major constituents in water samples (see Table 6). VUV, on the other hand, is strongly absorbed by anions commonly present in ground and surface water. For example, in the sample DW_highCl-, 90% of the VUV photons are absorbed by chloride (Cl^−^) in generating chlorine radicals (Cl^.^), owing to the high molar absorption coefficient of chloride ($\varepsilon_{VUV,Cl^{-}}=3800M.cm^{-1}$, (Furatian, 2017)). Cl^.^ undergoes many reactions, eventually to be scavenged or contributed to OMP degradation (Feng et al., 2007). Cl^.^ radicals are selective, but roughly equally reactive to electron-rich moieties compared to HO^.^ (Lei et al., 2019). Here, all VUV absorption by Cl^−^ is assumed to generate oxidative radicals equivalent to HO^.^, rendering AE_VUV_ equal to uAE_VUV_. Using the two aforementioned assumptions, uAE_UV_ and uAE_VUV_ are estimated. Note that a 1D graph representing log degradation of an OMP with respect to k’_total uAE_ instead of both k’_uAE,UV_ and k’_uAE,VUV_ has similar drawbacks as the use of k’_total AE_ (see Section 3.3). For example, the absolute log degradation of CBZ in DW_low_Cl- water matrix was 0.64 (1 cm cell) and 0.09 (2 cm cell) in VUV+UV/H_2_O_2_ and UV/H_2_O_2_ AOP, respectively. However, from Table 4, the ratio k’_VUV+UV/H2O2_ / k’_UV/H2O2_ AOP using k’_total uAE_ of CBZ in DW_low_Cl- water matrix is less than 1, implying that the degradation efficiency, per kWh.m ^−^ ^3^ addition, in a UV/H_2_O_2_ AOP is better than in a VUV+UV/H_2_O_2_ AOP. However, it should be noted that in reality no extra amount of energy is required for the addition of VUV. That is the pitfall of using k’_total uAE_. Therefore, a 2D plot that illustrates the dependence of log degradation on both uAE_UV_ and uAE_VUV_ is warranted.

**Table 6:** Molar absorption coefficients (M ^−^ ^1^.cm^−1^) and quantum yields of anions and cations commonly present in drinking and surface waters at UV and VUV.

Constituent	Molar absorption coefficient at UV	Quantum yield at UV	Molar absorption coefficient at VUV	Quantum yield at VUV
Na^+^	Negligible^1^^,^^2^		<0.01^1^	
K^+^			841^1^	
Ca^2^^+^			109^1^	
Cl^−^	Negligible^3^		3800±300^3^	0.42±0.02
Br^−^			12,000^4^	
I^−^	205.35 5		11,000 4	
SO_4_^2^^−^	<1 6		260^4^	0.64
NO_3_^−^	4 6	$\Phi_{HO^{.}}$= 0.09; pH=4–12^7^	5568^1^	
HCO_3_^−^	<0.01		269^1^	
CO_3_^2^^−^			1000 4	
Carbamazepine	6759±190	0.000067±0.00002		

1 (Duca et al., 2017).

2 (Birkmann et al., 2018).

3 (Furatian, 2017).

4 (Weeks et al., 1963).

5 (Awtrey and Connick, 1951).

6 (Buck et al., 1954).

7 (Mark et al., 1996).

#### 3.4.1 Development of 2D plots as reporting methodology for experimental data from the qCB

**Fig. 4:**
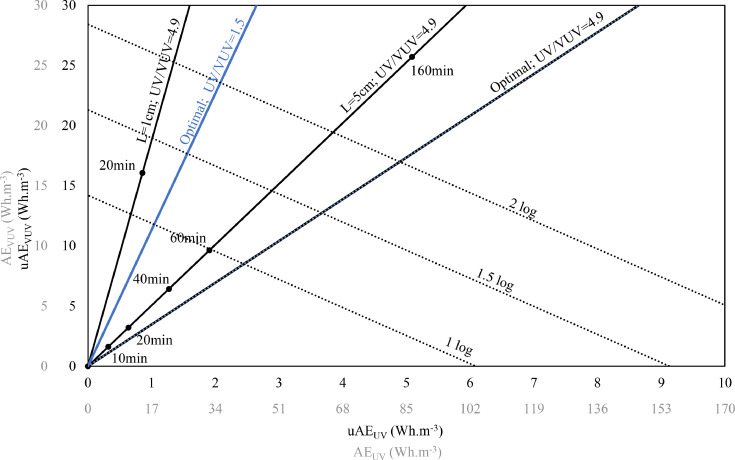
2D graph for degradation of CBZ in DW high Cl- matrix with H_2_O_2_ dosing of 10 mg.L ^−^ ^1^ (wherever applicable). The regression equation of the process is logdegradation = 0.164*uAE_UV_ + 0.0704*uAE_VUV_. The dotted lines and the solid lines indicate the isolog lines and design lines respectively. Optimal lines indicate the case of 100% utilization of the input UV and VUV for various electrical efficiency of lamp.

uAE_UV_ and uAE_VUV_ are calculated using the equations presented in Section 2.6.3. The effective use of the plot depends on the accuracy of the uAE_UV_ and uAE_VUV_ calculated. In this study, regression analysis was performed on experimental data from *q*CB system using the statistical software R (see SI on the generation of regression equations). Regression equations with log degradation as the output variable and uAE_VUV_ and uAE_UV_ as independent variables are further used to generate the 2D plots (Fig. 4, Fig. S4 and Fig. S5 in SI). The extreme points on the isolog lines (dotted lines in Fig. 4) on the x-axis and y-axis indicate the amount of uAE_UV_ and uAE_VUV_ required in a UV/H_2_O_2_ AOP and a standalone VUV+UV AOP, respectively, to achieve a given log degradation at a given H_2_O_2_ dosing. The points between the extremes on the isolog line indicate combinations of uAE_VUV_ and uAE_UV_ in VUV+UV/H_2_O_2_ AOP that will achieve a given log degradation. Note that uAE_VUV_ and uAE_UV_ are sufficient information for the replication of the experiment in a given water matrix for a specific OMP by a different laboratory independent on the design of the reactor used. It is crucial to consider that any conclusion that is derived from 2D plots is only valid for the specific H_2_O_2_ dosing used in the experiments whose experimental data were used to generate the regression equations.

#### 3.4.2 2D plots as a tool for predicting maximum possible degradation in pilot designs

Isolog lines indicates the maximum achievable log degradations of the specific OMP at the uAE_λ_ combination for the given water type and H_2_O_2_ concentration. This assertion is based on the assumption that the *q*CB system is a perfectly mixed system, thereby is a system in which the highest degradation of OMPs, at the given operational settings, can be achieved. In contrast, in pilot or full-scale reactors imperfect mixing is inevitable. By providing the maximum possible degradation, the 2D plot helps determine the window of hydrodynamic optimization that may be achieved through improving reactor design in pilot systems.

### 3.4.3 2D plots in designing of pilot-reactors

The design lines (solid lines that run through the origin in Fig. 4) indicate the effect of changing residence times and path length of the reactor on log degradation of an OMP. There are several implications of these design lines. The first is that, various combinations of uAE_VUV_ and uAE_UV_, at a specific H_2_O_2_ concentration, achievable in a reactor path length is deducible from the design lines without having to perform individual experiments. Subsequently, uAE_λ_ can be converted to the energy requirement (see Section 2.6.3) in individual cases to make an informed choice on the most energy efficient AOP. The ‘optimal’ design line indicates the situation where all the energy at UV and VUV generated from the lamp are absorbed by the sample. The optimal line in black corresponds to the output ratio of UV/VUV = 4.9 (lamp employed in the experiments presented in this paper) and the optimal line in blue corresponds to a hypothetical lamp with UV/VUV = 1.5. Thus, the effect of varying UV and VUV electrical efficiencies on the energy requirements of an AOP is deducible from the 2D plot. This is an extremely important outcome of the 2D plot and offers high degree of energy savings potential for the water industry.

The second implication is that these design lines help determine the maximum possible log degradation in a reactor configuration at a specific H_2_O_2_ concentration (see Section 3.4.2).

The third implication is that because each point on the design line corresponds to various residence times (i.e., flow in a reactor), the effect of varying flow on log degradations of OMPs at a specific H_2_O_2_ concentration can be deduced. It must be emphasized that when flow is varied in a reactor, the fluid hydrodynamics (and not only the residence time of the fluid) change as well. This could lead to enhanced mixing and better OMP degradations, however, the effect of change in flow on hydrodynamic and thereby on OMP degradations are not deducible from the 2D plot.

#### 3.4.4 Predicting energy requirements from the 2D plots

Energy requirement is a major factor in the selection of an AOP. It is a common practice that treatment plants mostly aim for a specified degradation rate (e.g., log removal) for a specific target OMP to assess the effectiveness of an AOP. 2D plots can be used to predict the energy required to degrade a specific OMP in a specific water matrix at a specific H_2_O_2_ concentration in various reactor configurations.

For example, from Fig. 4, to achieve 1-log degradation of CBZ in the DW_ high Cl- sample (dosed with 10 mg.L ^−^ ^1^ H_2_O_2_ wherever applicable; $a_{UV}=$0.056cm^−1^ and $a_{VUV}$=12.6cm^−1^), $uAE_{VUV}$ = 14.5 Wh.m ^−^ ^3^ and $uAE_{UV}$=6.2 Wh.m ^−^ ^3^ are required in a VUV+UV AOP and UV/H_2_O_2_ AOP respectively.

However, in VUV+UV/H_2_O_2_ AOP, the $uAE_{UV}$ required to achieve 1 log degradation is 2 Wh.m ^−^ ^3^ in a 5 cm cell. Assuming a lamp of $\eta_{\mathrm{UV}}$=33% and $\eta_{\mathrm{VUV}}$=6.7%, using Eq. (13), these uAE values are converted to E_EO_ values of 0.215 kWh.m ^−^ ^3^, 0.31 kWh.m ^−^ ^3^ and 0.1 kWh.m ^−^ ^3^ in VUV+UV AOP, UV/H_2_O_2_ AOP and VUV+UV/H_2_O_2_ AOP respectively. Fig. S4 and Fig. S5 in SI demonstrates the applicability of 2D plot for CBZ and ATZ in DW_low_Cl- water matrix respectively.

## 4 Conclusion

*q*CB systems have been widely used for laboratory-scale experimentation of AOPs. However, there is a need for standard approaches in the reporting and interpretation of *q*CB results for novel dual-wavelength AOPs. Also, currently there are scarce insights on using *q*CB data for design and development of pilot systems. The conventional approaches to report and compare *q*CB data, such as the apparent reaction rate constants based on time, dose and absorbed energy are either inadequate or inaccurate for the purpose of informing engineering feasibility and design.

Here, 2D plots based on useful absorbed energy is a new methodology introduced to serve as a standard methodology in reporting *q*CB data for dual-wavelength AOPs. The isolog lines on the plot indicates the uAE_UV_ and (or) uAE_VUV_ required to achieve a specified log degradation of a targeted OMP in a particular water matrix at a specific H_2_O_2_ dosing. The extremes of the 2D plot correspond to the standalone VUV and UV/H_2_O_2_ AOPs. It is thus possible to estimate the difference in energy investments required for the VUV+UV, UV/H_2_O_2_ and VUV+UV/H_2_O_2_ AOPs. These plots can also be used to predict the maximum possible degradation of an OMP in a specific water matrix and H_2_O_2_ concentration. Consequently, the optimization opportunity for design of a reactor with respect to mixing can be estimated. The design lines indicate the effect of varying path length and irradiation times (which can be translated to flow in a flow-through reactor), based on the relative and absolute contributions of uAE_UV_ and uAE_VUV_. The optimal design lines in the 2D plots opens the discussion of tailor-made lamps. Further, data from such plots can be used to approximate the E_EO_ required in pilot system achieve a desired OMP treatment.

## Declaration of Competing Interest

The authors declare that they have no known competing financial interests or personal relationships that could have appeared to influence the work reported in this paper.

## Data Availability

Data will be made available on request.

## Appendix Supplementary materials

Image, application 1
