# Supplementary material for: A novel approach to interpret quasi-collimated beam results to support design and scale-up of vacuum UV based AOPs
Source: Water Res X. 2022 Oct 9;17:100158. doi: 10.1016/j.wroa.2022.100158 (PMC9619181; doi:10.1016/j.wroa.2022.100158)
Supplement: Supplementary file 1 [file mmc1.docx]

**A novel approach to interpret quasi-collimated beam results to support design and scale-up of Vacuum UV based AOPs**

N. Kovoor George^1,2*^, B.A. Wols^2,3^, D. Santoro^4,5^, M. Borboudakis^2^, K. Bell^7^, W. Gernjak^8,9^

^1^ University of Girona, Plaça de Sant Domènec, 3, 17004 Girona, Spain

^2^ Wetsus, European Center of Excellence for Sustainable Water Technology, Oostergoweg 9, 8911MA Leeuwarden, the Netherlands

^3^KWR, Groningenhaven 7, 3433 PE Nieuwegein, the Netherlands

^4^Trojan Technologies, 3020 Gore Rd, London, ON N5V 4T7, Canada

^5^USP Technologies, 3020 Gore Rd, London, ON N5V 4T7, Canada

^6^PWNT water technology, Rijksweg 501, P.O Box 2046, 1990 AA Velserbroek, The Netherlands

^7^Brown and Caldwell, Walnut creek, California, United States

^8^Catalan Institute for Water Research (ICRA), 17003 Girona, Spain

^9^Catalan Institution for Research and Advanced Studies (ICREA), 08100 Barcelona, Spain

*Corresponding author: [nimmygeorge777@gmail.com](mailto:nimmygeorge777@gmail.com)

**Text S 1**

**Calculation of ratio of dose in 1 cm an 2 cm cells used in the collimated beam setup**

| ${D_{\lambda}= I}_{avg,\lambda}*t= (I_{0,\lambda}*RF*PF*DF)*\frac{\left( 1-{10}^{-a_{\lambda}l} \right)}{\ln\left( 10 \right)*a_{\lambda}*l}$ *t |  |  | (1) |
| --- | --- | --- | --- |

| $\frac{D_{2cm}}{D_{1cm}}=\frac{{RF}_{2cm}* {PF}_{2cm}*{DF}_{2cm}*\left( 1-{10}^{-a_{\lambda}*2} \right)_{2cm}}{2}* \frac{1}{{{RF}_{1cm}* {PF}_{1cm}*{DF}_{1cm}*\left( 1-{10}^{-a_{\lambda}*1} \right)_{1cm}}}$ |  |  | (2) |
| --- | --- | --- | --- |

The ratio $\left[ \frac{{RF}_{2cm}* {PF}_{2cm}*{DF}_{2cm}}{{RF}_{1cm}* {PF}_{1cm}*{DF}_{1cm}} \right]_{UV}$and $\left[ \frac{{RF}_{2cm}* {PF}_{2cm}*{DF}_{2cm}}{{RF}_{1cm}* {PF}_{1cm}*{DF}_{1cm}} \right]_{VUV}$ is 0.971 for the different water types. Using the absorbances of various water types, the ratio $\left[ \frac{D_{2cm}}{D_{1cm}} \right]_{UV}$and $\left[ \frac{D_{2cm}}{D_{1cm}} \right]_{VUV}$are calculated in Table

Table S 1: Absorbances of various water samples and their $\left[ \frac{D_{2cm}}{D_{1cm}} \right]_{\mathrm{UV}}$and $\left[ \frac{D_{2cm}}{D_{1cm}} \right]_{\mathrm{VUV}}$ratios

|  | MilliQ® | RO | DW_high Cl- | DW_Low Cl- | SWWE |
| --- | --- | --- | --- | --- | --- |
| $a_{UV} ({cm}^{-1})$ | 0.006 | 0.006 | 0.056 | 0.055 | 0.535 |
| $a_{VUV} ({cm}^{-1})$ | 1.8 | 5.7 | 12.6 | 4.04 | 11.9 |
| $\left[ \frac{D_{2cm}}{D_{1cm}} \right]_{\mathrm{UV}}$ | 0.96 | 0.96 | 0.91 | 0.91 | 0.63 |
| $\left[ \frac{D_{2cm}}{D_{1cm}} \right]_{\mathrm{VUV}}$ | 0.49 | 0.49 | 0.49 | 0.49 | 0.49 |

Note that these calculations assume that the incident radiation $I_{0,UV}$ and $I_{0,VUV}$ remains the same for 1cm and 2 cm cell. For example, the $\frac{I_{0,UV,2cm}}{I_{0,UV,1cm}}$ for the experiments with MilliQ^®^ was 0.9, thereby rendering the $\left[ \frac{D_{2cm}}{D_{1cm}} \right]_{\mathrm{UV}}$to 0.86 (i.e, 0.96*0.90)

**Text S 2**

**Generation of regression equations from qCB experimental data**

The degradation rate of an OMP via the ${HO}^{.}$ is given by:

| $\frac{d[C]}{dt}=k_{C,{HO}^{.}}*{[HO}^{.}]*[C]$ |  |  | (3) |
| --- | --- | --- | --- |

where $[C]$ is the concentration of the OMP in M (mols.L^-1^); $t$ is the time in s; $k_{C,{HO}^{.}}$ is the second-order reaction rate constant between ${HO}^{.}$ and the OMP in M^-1^.s^-1^; and ${[HO}^{.}]$ is the concentration of hydroxyl radicals in M.

The general rate equation for the consumption of ${HO}^{.}$ is given by:

| $\frac{d[S_{i}]}{dt}=\sum_{i=1}^{n} k_{S_{i},{HO}^{.}}*{[HO}^{.}]*[S_{i}]$ |  |  | (4) |
| --- | --- | --- | --- |

where $[S_{i}]$ is the concentration of constituent in M; $k_{S_{i},{HO}^{.}}$ is the second-order reaction rate constant between ${HO}^{.}$ and the constituent in M^-1^.s^-1^ and $n$ is the total number of constituents that react with ${HO}^{.}$. ${[HO}^{.}]$ is consumed by OMPs and the scavenging due to background matrix in a sample.

Integrated form of Eq. 3 results in:

| $ln\left( \frac{{[C]}_{t}}{{[C]}_{0}} \right)={-k}_{C,{HO}^{.}}*{[HO}^{.}]*t$ = $-k^{'}*t$ |  |  | (5) |
| --- | --- | --- | --- |

Generation of ${HO}^{.}$ in VUV+UV/H_2_O_2_ AOP takes place through the following two primary pathways.

| $H_{2}O_{2}+h\nu_{\mathrm{UV}}\to2HO^{.}$ | $\Phi_{UV,H_{2}O_{2}}=1$ |  | (6) |
| --- | --- | --- | --- |
| $H_{2}O+h\nu_{\mathrm{VUV}}\to HO^{.}+ H^{.}$ | $\Phi_{VUV1,H_{2}O}=0.33$ |  | (7) |

Additionally, ${HO}^{.}$ is generated via:

| $H_{2}O+h\nu_{\mathrm{VUV}}\to HO^{.}+ H^{+}+ e_{\mathrm{aq}}^{-}$ | $\Phi_{VUV2,H_{2}O}=0.045$ |  | (8) |
| --- | --- | --- | --- |
| $H_{2}O_{2}+h\nu_{\mathrm{VUV}}\to2HO^{.}$ | $\Phi_{VUV,H_{2}O_{2}}=1$ |  | (9) |

The term $h\nu_{\mathrm{UV}}$ and $h\nu_{\mathrm{VUV}}$ must be quantified. The first law of photochemistry states that only the light which is absorbed by a molecule can be effective in producing photochemical change in the molecule. In a qCB, the rate of photons entering a sample solution can be determined using a suitable radiometer and detector combination or using actinometry experiments. The quantity determined is called incident irradiance, $I_{0,\lambda}$ (W.cm^-2^). Irradiance can be converted in to the photons entering the sample using the J.einstein^-1^ (note: 1 mol of photon=1 einstein) at that particular wavelength. The amount of irradiance that is absorbed by the sample at a given wavelength, $I_{abs,\lambda}$ (W.cm^-2^) depends on the absorbance, $a$ (cm^-1^) and path length (cm) of the sample via the Beer-Lambert’s law.

| $I_{abs,\lambda}=$ $I_{0,\lambda}*\left( 1-{10}^{-a_{\lambda}l} \right)$ |  |  | (10) |
| --- | --- | --- | --- |

In a qCB setup, the $I_{0,\lambda}$ must be multiplied with the reflection factor (RF), petri factor (PF) and divergence factor (DF) to get the accurate $I_{abs,\lambda}$. Eq. 10 will be modified to:

| $I_{abs,\lambda}=$ $I_{0,\lambda}*\left( 1-{10}^{-a_{\lambda}l} \right)*RF*PF*DF$ |  |  | (11) |
| --- | --- | --- | --- |

Therefore, the total absorbed energy, ${AE}_{\lambda}$ (Wh.cm^-3^) over a time, $t$ (s) is given by:

| ${AE}_{\lambda}=\frac{I_{abs,\lambda}}{3600}*\frac{S}{V}*t$ | $Type equation here.$ |  | (12) |
| --- | --- | --- | --- |

where, $S$ is the surface area of the sample irradiated in cm^2^; $V$ is the volume of the sample irradiated in cm^3^. (Note that $\frac{S}{V}$ is equal to the pathlength $l$ of the sample in case of the cylindrical cell used in this study).The factor $\frac{1}{3600}$ converts J.cm^-2^ to Wh.cm^-2^.

Only a fraction of the ${AE}_{\lambda}$ is absorbed by constituents that contribute towards the generation of $HO^{.}$ that are indicated in Eq.6, 7, 8 and 9. The rest of the photons are scavenged by the background constituents of the sample. These fractions can be expressed as:

| $f_{UV,H_{2}O_{2}}= \frac{\varepsilon_{H_{2}O_{2},UV}*C_{H_{2}O_{2}}}{\sum_{i}^{n} \varepsilon_{i,UV}{*C}_{i}}$ |  |  | (13) |
| --- | --- | --- | --- |
| $f_{UV, x}=\frac{\varepsilon_{x,UV}{*C}_{x}}{\sum_{i}^{n} \varepsilon_{i,UV}*C_{i}}$ |  |  | (14) |
| $f_{VUV,H_{2}O}=\frac{\varepsilon_{H_{2}O,VUV}*C_{H_{2}O}}{\sum_{i}^{n} \varepsilon_{i,VUV}*C_{i}}$ |  |  | (15) |
| $f_{VUV,H_{2}O_{2}}=\frac{\varepsilon_{H_{2}O_{2},VUV}*C_{H_{2}O_{2}}}{\sum_{i}^{n} \varepsilon_{i,VUV}*C_{i}}$ |  |  | (16) |
| $f_{VUV, x}=\frac{\varepsilon_{x,VUV}{*C}_{x}}{\sum_{i}^{n} \varepsilon_{i,VUV}*C_{i}}$ |  |  | (17) |

where is $\varepsilon_{i,\lambda}$ the molar absorbance of the constituent $i$ in the sample at a given wavelength in M^-1^.m^-1^ and $C$ is its respective concentration in M. The term $\varepsilon_{x,\lambda}C_{x}$is the fraction absorbed by any constituent $x$ in the sample that can generate radicals at a given $\lambda$.; $n$ is the number of constituents in the sample.

Note that H_2_O_2_ will in most cases be the only constituent that contributes towards generation of radicals on absorption of UV (i.e, $f_{UV, x}=0$). However, besides H_2_O and H_2_O_2_, some anions like chloride (Cl^-^) absorbs VUV to generate radicals. For the sake of simplicity, all the ${AE}_{VUV}$ is assumed to be absorbed by sample constituents that generates radicals resulting in Eq. 19.

A product of the fractions in Eq. 13 and 15 and the ${AE}_{\lambda}$ gives useful absorbed energy, ${uAE}_{\lambda}$ (Wh.cm^-3^).

| ${uAE}_{UV}={AE}_{UV}*f_{UV,H_{2}O_{2}}$ |  |  | (18) |
| --- | --- | --- | --- |
| ${uAE}_{VUV}={AE}_{VUV}$ |  |  | (19) |

The rate of $HO^{.}$ generated per photon of $\lambda$ absorbed by $H_{2}O_{2}$ or $H_{2}O$ by depends on the quantum yield of the process, $\Phi$. Implementing $\Phi$ in Eq. 18 and 19

| $\frac{d{[HO^{.}]}_{VUV}}{dt}=$ $\frac{{uAE}_{UV}*3600*\Phi_{UV,H_{2}O_{2}}}{U_{UV}*t}=\frac{{0.0076*uAE}_{UV}}{t}$ |  |  | (20) |
| --- | --- | --- | --- |
| $\frac{d{[HO^{.}]}_{UV}}{dt}=$ $\frac{{uAE}_{VUV}*3600*{(\Phi}_{VUV1,H_{2}O}+\Phi_{VUV2,H_{2}O})}{U_{VUV}*t}=\frac{{0.0021*uAE}_{VUV}}{t}$ |  |  | (21) |
| $\frac{d{[HO^{.}]}_{total}}{dt}=\frac{d{[HO^{.}]}_{UV}}{dt}+\frac{d{[HO^{.}]}_{VUV}}{dt}$ |  |  | (22) |

Note that here Eq. 20 and 21 refers to the $[HO^{.}]$ generation rates in UV/H_2_O_2_ AOP and VUV AOP, respectively and Eq.22 refers to the $[HO^{.}]$ generation rates in VUV+UV/H_2_O_2_ AOP

Owing to its high reactivity with OMPs as wells as the background scavenging constituents in samples, $HO^{.}$ can be assumed to reach steady-state concentrations within very short time. In such situation, $[HO^{.}]$ can be substituted by ${{[HO}^{.}]}_{ss}$ in Eq. 3,4 and 5.

In a UV/H_2_O_2_ AOP, on equating the $[HO^{.}]$ generation rate (Eq. 20) and the $[HO^{.}]$ consumption rate Eq. 4, the ${{[HO}^{.}]}_{ss}$ is obtained as following:

| ${{[HO}^{.}]}_{ss}=\frac{{0.0076*uAE}_{UV}}{t*\sum_{i=1}^{n} k_{S_{i},{HO}^{.}}*\left[ S_{i} \right]}$ |  |  | (23) |
| --- | --- | --- | --- |

In a VUV AOP, on equating the $[HO^{.}]$ generation rate (Eq.21) and the $[HO^{.}]$ consumption rate Eq. 4, the ${{[HO}^{.}]}_{ss}$ is obtained as following:

| ${{[HO}^{.}]}_{ss}=\frac{{0.0021*uAE}_{VUV}}{t*\sum_{i=1}^{n} k_{S_{i},{HO}^{.}}*\left[ S_{i} \right]}$ |  |  | (24) |
| --- | --- | --- | --- |

Finally, in a VUV+UV/H_2_O_2_ AOP, on equating the $[HO^{.}]$ generation rate (Eq. 22) and the $[HO^{.}]$ consumption rate Eq. 4, the ${{[HO}^{.}]}_{ss}$ is obtained as following:

| ${{[HO}^{.}]}_{ss}=\frac{{0.0076*uAE}_{UV}+{0.0021*uAE}_{VUV}}{t*\sum_{i=1}^{n} k_{S_{i},{HO}^{.}}*\left[ S_{i} \right]}$ |  |  | (25) |
| --- | --- | --- | --- |

Substituting Eq. 23, 24 and 25 in Eq. 5, the degradation of the target OMP in a UV/H_2_O_2_ AOP, VUV AOP and VUV+ UV/H_2_O_2_ AOP are respectively obtained in terms of ${uAE}_{\lambda}$ and scavenging capacity of a sample.

| $ln\left( \frac{{[C]}_{t}}{{[C]}_{0}} \right)=\frac{{{-k}_{C,{HO}^{.}}* 0.0076*uAE}_{UV}}{\sum_{i=1}^{n} k_{S_{i},{HO}^{.}}*\left[ S_{i} \right]}$ |  |  | (26) |
| --- | --- | --- | --- |
| $ln\left( \frac{{[C]}_{t}}{{[C]}_{0}} \right)=\frac{{{-k}_{C,{HO}^{.}}* 0.0021*uAE}_{VUV}}{\sum_{i=1}^{n} k_{S_{i},{HO}^{.}}*\left[ S_{i} \right]}$ |  |  | (27) |
| $ln\left( \frac{{[C]}_{t}}{{[C]}_{0}} \right)={-k}_{C,{HO}^{.}}*\left( \frac{{0.0076*uAE}_{UV}+{0.0021*uAE}_{VUV}}{\sum_{i=1}^{n} k_{S_{i},{HO}^{.}}*\left[ S_{i} \right]} \right)$ |  |  | (28) |

Regression equations are generated from feeding the experimental data into commercial software R and takes the form:

| $ln\left( \frac{{[C]}_{t}}{{[C]}_{0}} \right)=x{*uAE}_{UV}+ y{*uAE}_{VUV}$ |  |  | (29) |
| --- | --- | --- | --- |

Table S 2: k’_time_, k’_Total dose_ , k’_Total AE_ and k’_Total uAE_ for ATZ, CBZ, MTF and n-PBN in RO, DW_high Cl-, DW_low Cl- and SWWE water matrices.

|  | **UV/H2O2** | | | | **VUV+UV** | | | | **VUV+UV/H2O2** | | | |
| --- | --- | --- | --- | --- | --- | --- | --- | --- | --- | --- | --- | --- |
|  | **ATZ** | **CBZ** | **MTF** | **n-PBN** | **ATZ** | **CBZ** | **MTF** | **n-PBN** | **ATZ** | **CBZ** | **MTF** | **n-PBN** |
| **RO** |  |  |  |  |  |  |  |  |  |  |  |  |
| k_time_ (s^-1^) | 6.1E-04 | 1.6E-03 | 1.7E-04 | 1.4E-03 | 2.8E-03 | 8.1E-03 | 6.4E-04 | 8.7E-03 | 2.5E-03 | 8.4E-03 | 9.9E-04 | 7.4E-03 |
| k_total dose_ (mJ^-1^.cm^-1^) | 3.0E-03 | 7.7E-03 | 8.3E-04 | 6.9E-03 | 1.1E-02 | 3.1E-02 | 2.5E-03 | 3.3E-02 | 9.8E-03 | 3.3E-02 | 3.8E-03 | 2.9E-02 |
| k_total AE_ (W^-1^h^-1^.m^3^) | 7.0E-01 | 1.8E+00 | 1.9E-01 | 1.6E+00 | 1.7E-01 | 5.0E-01 | 4.0E-02 | 5.4E-01 | 1.5E-01 | 5.0E-01 | 5.8E-02 | 4.4E-01 |
| k_total uAE_ (W^-1^h^-1^.m^3^) | 8.1E-01 | 2.1E+00 | 2.2E-01 | 1.8E+00 | 1.7E-01 | 5.1E-01 | 4.1E-02 | 5.5E-01 | 1.5E-01 | 5.0E-01 | 5.9E-02 | 4.4E-01 |
|  |  |  |  |  |  |  |  |  |  |  |  |  |
| **DW_high_Cl-** |  |  |  |  |  |  |  |  |  |  |  |  |
| k_time_ (s^-1^) | 2.8E-04 | 3.4E-04 | 6.7E-05 | 2.0E-04 | 4.1E-04 | 1.1E-03 | 1.2E-04 | 2.1E-03 | 5.0E-04 | 1.5E-03 | 2.2E-04 | 1.8E-03 |
| k_total dose_ (mJ^-1^.cm^-1^) | 1.4E-03 | 1.7E-03 | 3.3E-04 | 9.9E-04 | 2.0E-03 | 5.5E-03 | 5.9E-04 | 1.0E-02 | 2.5E-03 | 7.2E-03 | 1.1E-03 | 8.7E-03 |
| k_total AE_ (W^-1^h^-1^.m^3^) | 3.1E-02 | 3.7E-02 | 7.4E-03 | 2.2E-02 | 2.7E-02 | 7.4E-02 | 8.1E-03 | 1.4E-01 | 3.2E-02 | 9.2E-02 | 1.4E-02 | 1.1E-01 |
| k_total uAE_ (W^-1^h^-1^.m^3^) | 3.2E-01 | 3.8E-01 | 7.6E-02 | 2.3E-01 | 6.1E-02 | 1.7E-01 | 1.8E-02 | 3.1E-01 | 6.6E-02 | 1.9E-01 | 2.9E-02 | 2.3E-01 |
|  |  |  |  |  |  |  |  |  |  |  |  |  |
| **DW_low_cl-** |  |  |  |  |  |  |  |  |  |  |  |  |
| k_time_ (s^-1^) | 2.5E-04 | 3.2E-04 | 2.8E-05 | 2.4E-04 | 7.0E-04 | 1.6E-03 | 2.6E-04 | 9.4E-04 | 9.4E-04 | 2.4E-03 | 3.3E-04 | 1.5E-03 |
| k_total dose_ (mJ^-1^.cm^-1^) | 1.4E-03 | 1.8E-03 | 1.6E-04 | 1.3E-03 | 2.8E-03 | 6.3E-03 | 1.0E-03 | 3.8E-03 | 3.8E-03 | 9.8E-03 | 1.3E-03 | 6.2E-03 |
| k_total AE_ (W^-1^h^-1^.m^3^) | 3.9E-02 | 5.1E-02 | 4.5E-03 | 3.8E-02 | 3.0E-02 | 6.7E-02 | 1.1E-02 | 4.0E-02 | 3.9E-02 | 9.9E-02 | 1.3E-02 | 6.3E-02 |
| k_total uAE_ (W^-1^h^-1^.m^3^) | 3.2E-01 | 4.3E-01 | 3.7E-02 | 3.2E-01 | 4.5E-02 | 9.9E-02 | 1.6E-02 | 6.0E-02 | 5.6E-02 | 1.4E-01 | 1.9E-02 | 9.1E-02 |
|  |  |  |  |  |  |  |  |  |  |  |  |  |
| **SWWE** |  |  |  |  |  |  |  |  |  |  |  |  |
| k_time_ (s^-1^) | 7.5E-05 | 3.4E-06 | 2.8E-06 | 1.4E-05 | 1.7E-04 | 4.6E-04 | 2.5E-05 | 8.7E-05 | 1.9E-04 | 2.1E-04 | 2.7E-05 | 1.1E-04 |
| k_total dose_ (mJ^-1^.cm^-1^) | 9.8E-04 | 4.4E-05 | 3.7E-05 | 1.9E-04 | 1.2E-03 | 3.0E-03 | 1.7E-04 | 5.8E-04 | 1.3E-03 | 1.4E-03 | 1.8E-04 | 7.1E-04 |
| k_total AE_ (W^-1^h^-1^.m^3^) | 2.9E-03 | 1.3E-04 | 1.1E-04 | 5.5E-04 | 2.6E-03 | 6.9E-03 | 3.8E-04 | 1.3E-03 | 2.9E-03 | 3.2E-03 | 4.0E-04 | 1.6E-03 |
| k_total uAE_ (W^-1^h^-1^.m^3^) | 9.8E-02 | 4.4E-03 | 3.7E-03 | 1.9E-02 | 1.1E-02 | 2.9E-02 | 1.6E-03 | 5.5E-03 | 1.1E-02 | 1.3E-02 | 1.6E-03 | 6.3E-03 |

N_2_

Collimator

Stir bar

Sample holder

Lamp Housing

UV lamp

Water sample

Stir plate

Figure S 1: Quasi-collimated beam setup.

Figure S 2: Dependence on D_UV_ and D_VUV_ on the path length of the reactor at various water absorbance.

Figure S 3: Dependence on AE_UV_ and AE_VUV_ on the path length of the reactor at various water absorbance.

Figure S 4: 2D graph for degradation of CBZ in DW low Cl- matrix at [H_2_O_2_]=10mg.L^-1^. The regression equation of the process is log degradation = 0.27*uAE_UV_ + 0.046*uAE_VUV_. The dotted lines and the solid lines indicate the iso-log lines and design lines respectively. Optimal lines indicate the case of 100% utilization of the input UV and VUV for various electrical efficiency of lamp.

Figure S 5: 2D graph for degradation of ATZ in DW low Cl- matrix at [H_2_O_2_]=10 mg.L^-1^. The regression equation of the process is log degradation = 0.079*uAE_UV_ + 0.008*uAE_VUV_. The dotted lines and the solid lines indicate the iso-log lines and design lines respectively. Optimal lines indicate the case of 100% utilization of the input UV and VUV for various electrical efficiency of lamp.
